# The level of active DNA demethylation compounds in leukocytes and urine samples as potential epigenetic biomarkers in breast cancer patients

**DOI:** 10.1038/s41598-024-56326-5

**Published:** 2024-03-18

**Authors:** Kinga Linowiecka, Jolanta Guz, Tomasz Dziaman, Olga Urbanowska–Domańska, Ewelina Zarakowska, Anna Szpila, Justyna Szpotan, Aleksandra Skalska-Bugała, Paweł Mijewski, Agnieszka Siomek-Górecka, Rafał Różalski, Daniel Gackowski, Ryszard Oliński, Marek Foksiński

**Affiliations:** 1 Department of Clinical Biochemistry, Faculty of Pharmacy, Collegium Medicum in Bydgoszcz, Nicolaus Copernicus University in Toruń, Karlowicza 24, 85‑092 Bydgoszcz, Poland; 2 Department of Human Biology, Institute of Biology, Faculty of Biological and Veterinary Sciences, Nicolaus Copernicus University in Toruń, Lwowska 1, 87-100 Toruń, Poland; 3 Department of Oncology, Professor Franciszek Lukaszczyk Oncology Centre, Romanowskiej 2, 85-796 Bydgoszcz, Poland

**Keywords:** Breast cancer, DNA demethylation, TET3, 5-methylcytosine, 5-hydroxymethylocytosine, Vitamin C, SLC23A1, SLC23A2, Breast cancer, Tumour biomarkers

## Abstract

The active DNA demethylation process, which involves TET proteins, can affect DNA methylation pattern. TET dependent demethylation results in DNA hypomethylation by oxidation 5-methylcytosine (5-mC) to 5-hydroxymethylcytosine (5-hmC) and its derivatives. Moreover, TETs’ activity may be upregulated by ascorbate. Given that aberrant DNA methylation of genes implicated in breast carcinogenesis may be involved in tumor progression, we wanted to determine whether breast cancer patients exert changes in the active DNA demethylation process. The study included blood samples from breast cancer patients (n = 74) and healthy subjects (n = 71). We analyzed the expression of genes involved in the active demethylation process (qRT-PCR), and 5–mC and its derivatives level (2D-UPLC MS/MS). The ascorbate level was determined using UPLC-MS. Breast cancer patients had significantly higher *TET3* expression level, lower 5-mC and 5-hmC DNA levels. *TET3* was significantly increased in luminal B breast cancer patients with expression of hormone receptors. Moreover, the ascorbate level in the plasma of breast cancer patients was decreased with the accompanying increase of sodium-dependent vitamin C transporters (*SLC23A1* and *SLC23A2*). The presented study indicates the role of TET3 in DNA demethylation in breast carcinogenesis.

## Introduction

The molecular basis of breast cancer is not entirely investigated. Considering recent discoveries, the initiation of breast carcinogenesis, similarly to the other malignancies, is linked with altered genes’ function, which may lead to malignant transformation. The epigenetic control of gene expression mainly refers to structural DNA modifications or chromatin organization, which can modulate the transcription process^1^. The most fundamental and widely described epigenetic modification is DNA methylation which occurs mainly in CpG dinucleotides^2^. The product of DNA methylation: 5-methylcytosine (5-mC) is arranged into the regulation of gene expression and thus organismal fate.

The DNA methylation pattern is not only dependent on methyl group transfer but also on active DNA demethylation. The mechanism of active DNA demethylation involves TET (ten eleven translocation) family proteins^3^, which are arranged in the hydroxylation of 5-mC to 5-hydroxymethylcytosine (5-hmC), and further into 5-formylcytosine (5-fC), and 5-caroboxylcytosine (5-caC)^3,4^. The last two modifications are finally excised by thymine DNA glycosylase (TDG) in the base excision repair process (BER)^5^.

The global DNA hypomethylation is a distinctive hallmark of cancer, including breast malignancy^6,7^. The loss of DNA methylation marks is frequently more apparent in tumor progression, in advanced stages of the disease^8–12^. Nevertheless, DNA hypomethylation may be also a phenomenon detected in the early stages of carcinogenesis^13–16^. Moreover, the loss of the other cytosine derivative: 5-hmC is equally reported in human cancers, including breast cancer cells^17,18^. Recent developments in this field have revealed that the low level of 5-hmC is frequently associated with low 5-mC content in cancer epigenome^19–21^. Therefore, the role of 5-hmC has risen to serve as a biomarker of malignant transformation, which is an important component in assessing the active DNA demethylation process.

Apart from global DNA hypomethylation, hypermethylation of CpG islands of promoter regions genes plays a key role in cancer cells. The extensive research on breast cancer cell lines and breast tumors revealed almost 100 genes that are hypermethylated in breast cancer^7^. A great majority of them are involved in cellular signaling, cell cycle regulation, DNA repair, metastasis, and tissue infiltration^22^.

The fluctuations of 5-mC and its derivatives levels are predominantly involved with altered TETs activity. TETs mutations were detected in leukemia^23,24^, colon cancer, lung cancer, bladder cancer, or melanoma^25^. So far, however, no TETs’ mutation has been found in breast cancer. Although the decrease of 5-hmC is frequently observed in cancers, regardless of verified TETs’ mutations, it may be involved with altered gene expression of TETs. The level of TETs expression varies depending on cancer type: it has been investigated that expression of all TETs is decreased in colon cancer^23,32^, cervical cancer^26^ or melanoma^27^, but in gastric cancer^28,29^, hepatocellular cancer^30^, prostate cancer^31^ or gliomas^32^ only reduction of TET1 expression has been reported. Consequently, it seems that the identification of a particular TET protein which may be potentially involved in breast carcinogenesis may be crucial for understanding the underlying mechanism of breast cancer initiation and progression.

Moreover, vitamin C (ascorbic acid, AA) plays a key role in restoring activity of TET enzymes. Since ascorbic acid is known as a significant reducing factor, it can easily restore the oxidation state of iron ion in the catalytic center of TET proteins^33^. Ascorbic acid absorption is executed using active transport via sodium-dependent vitamin C transporters namely SVCT1 and SVCT2 which are products of different genes, *SLC23A1* and *SLC23A2*, respectively^34–36^. There is a relationship between vitamin C level, its transporters expression, and cancer initiation^37–42^. It seems plausible that vitamin C may not only exert beneficial outcome for diminishing the side effects of chemotherapy or radiotherapy^43–46^, but since its level correlates with 5-hmC^20^, it also may impact on regulation of gene expression.

Recent years have provided us with a considerable amount of literature on levels of 5-mC and 5-hmC in cancer tissues^47–50^, demonstrating their importance as reliable cancer biomarkers. However, as tumor-lesioned tissues may be not easily obtained, we raised a question whether changes in the active DNA demethylation process are only defined to breast cancer tissues, or are global phenomena which also occur in surrogate tissues. Leukocytes can serve as reliable “carriers” of information, reflecting the changes in tissue milieu, specifically environmentally-induced DNA alterations^20,51,52^. Moreover, it should be noted that during the active DNA demethylation process, after elimination by BER mechanism, modified deoxynucleosides and nucleobases appear in the bloodstream, followed by their elimination in the urine^53,54^. Therefore, the paper focuses on the examination of breast cancer surrogate tissues – leukocytes as well as urine as a source of metabolic elimination products. We analyzed the expression level of genes involved in active DNA demethylation *(TET1*, *TET2*, *TET3*, *TDG*) as well as in ascorbic acid transport (*SLC23A1* and *SLC23A2*) in leukocytes, and the level of 5-mC and its derivatives in leukocytes and urine samples from breast cancer patients qualified to the study. In addition to epigenetic modifications of DNA, we also determined vitamin C level in blood plasma, as well as in leukocytes. To our knowledge, it is the first study that undergoes comprehensive analyses of the active DNA demethylation process in breast cancer.

## Results

### Significant increase of TET3 mRNA expression in blood samples from breast cancer patients with moderately differentiated tumor (G2)

**Figure 1:**
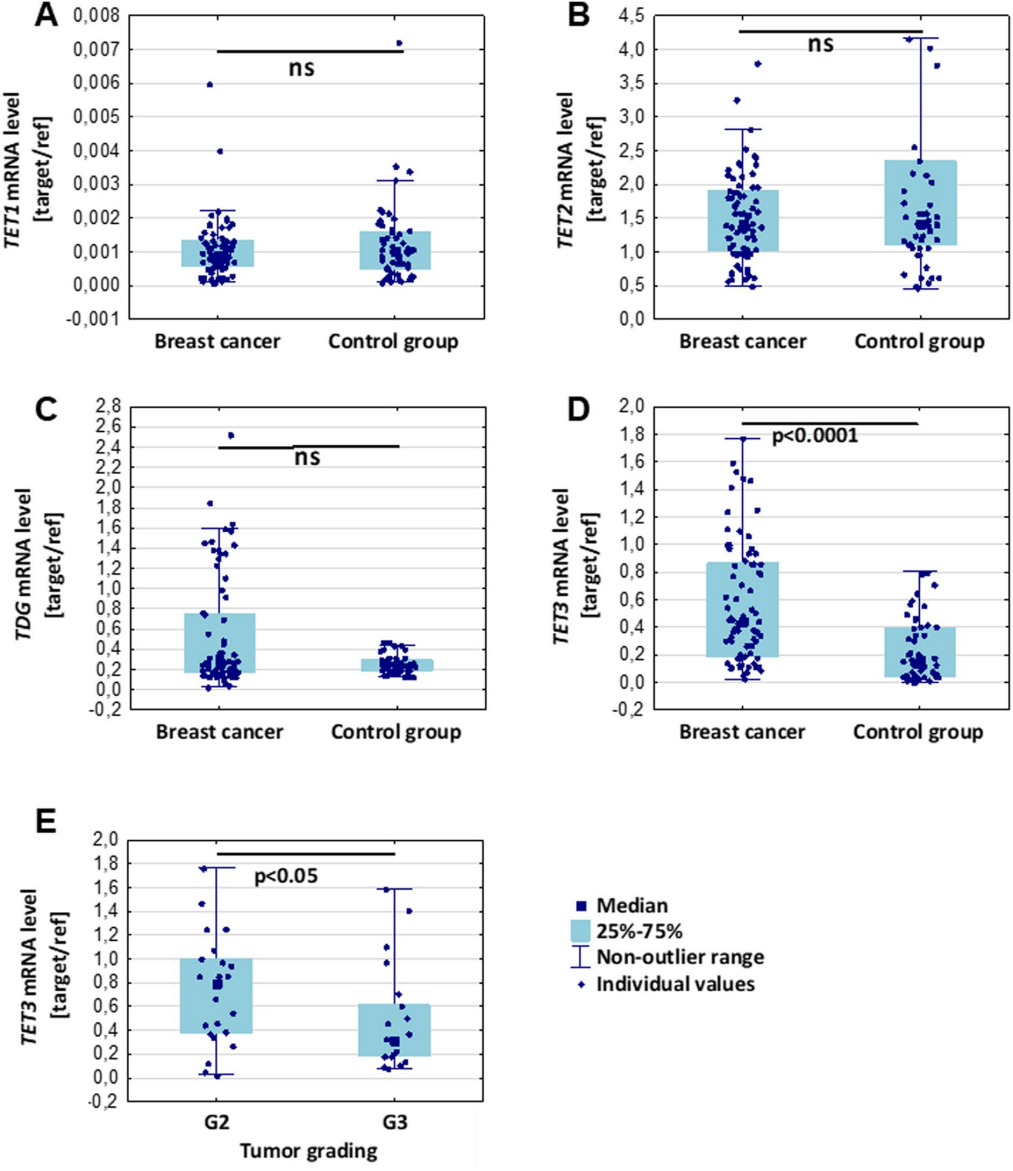
Comparison of TET1, TET2, TET3, and TDG mRNA expression between leukocytes from breast cancer patients and the control group. Expression levels of TET1 (**A**), TET2 (**B**), and TDG (**C**) were not statistically different between breast cancer and control groups. TET3 mRNA expression level was significantly increased in breast cancer patients (**D**), and in grade 2 breast tumors (**E**). Expression level was presented as a relative value normalized to *ACTB* (β–actin gene) and *G6PD* (glucose–6–phosphate dehydrogenase gene). ns – non-statistical difference.

Our present study involved for the first time the global observation of changes in the expression level of TETs and the levels of 5-mC and its derivatives in leukocytes from breast cancer women. We found increase of TET3 (*p* < 0.001) expression level in leukocytes of breast cancer group in comparison to healthy subjects (Fig. 1D). Interestingly, we found also that expression of TET3 is significantly higher in leukocytes from patients with grade G2 (moderately differentiated tumor) than G3 (poorly differentiated tumor) (*p* < 0.05) (Fig. 1E). Breast cancer patients did not significantly differ from control group in terms of TET1, TET2, and TDG mRNA expression (Fig. 1A–C).

### Decreased level of 5-hmC in leukocytes along with the increased level of 5-caC in the urine of breast cancer patients

**Figure 2:**
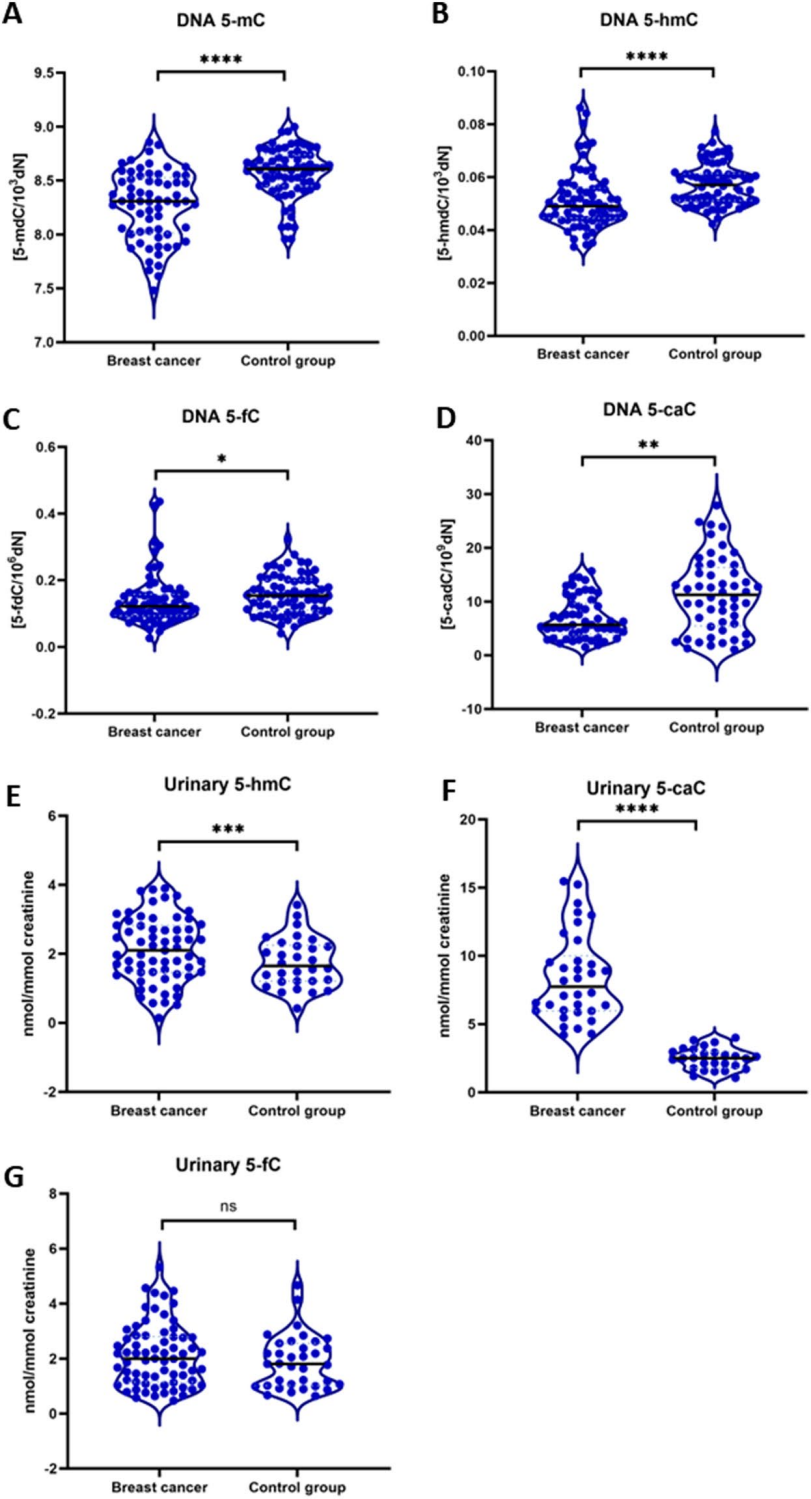
Comparison of 5-mC, 5-hmC, 5-fC, and 5-caC in the blood (DNA isolated from leukocytes) (**A**, **B**, **C**, **D**) and in urine (**E**, **F**, **G**) samples of breast cancer patients and the control group. Breast cancer leukocytes showed a significant decrease of 5-mC (**A**), 5-hmC (**B**), 5-fC (**C**), and 5-caC (**D**) in comparison to the control group. 5-mC and its derivatives levels were presented as a level of modified bases to unmodified deoxynucleosides, which was expressed as a number of modified molecules per 10^–3^ (for 5-mC and 5-hmC), 10^–6^ (for 5-fC) or 10^–9^ (for 5-caC) of unmodified deoxynucleosides. In urine samples, 5-hmC (**E**) and 5-caC (**F**) were significantly increased in breast cancer samples, whereas 5-fC (**G**) did not change significantly between these two groups. 5-hmC and its derivatives in urine were presented as a relative value based on the concentration of urinary creatinine. *p < 0.05; **p < 0.01; ***p < 0.001; *p* < 0.0001; ns-non – statistical difference.

Patients with breast cancer presented significantly lower levels of 5-mC in leukocytes’ DNA (*p* < 0.0001) in comparison to controls. Furthermore, in the leukocytes of those patients, we noticed also a decreased level of 5-hmC (*p* < 0.0001) (Fig. 2A–D). The lower level was also observed in terms of 5-fC and 5-caC in the breast cancer group in comparison to healthy subjects.

In urine samples (Fig. 2E–G) we observed significantly higher levels of 5-hmC (*p* < 0.001) in breast cancer samples. Correspondingly to DNA of leukocytes, 5-caC level in urine was also elevated in the breast cancer group (*p* < 0.0001). We also found a significantly lower level of 5-fC in urine samples of breast cancer patients in comparison to the control group (*p* < 0.05).

### Breast cancer patients display a decrease in vitamin C in plasma, and an increase in SLC23A1 and SLC23A2 expression in leukocytes

**Figure 3:**
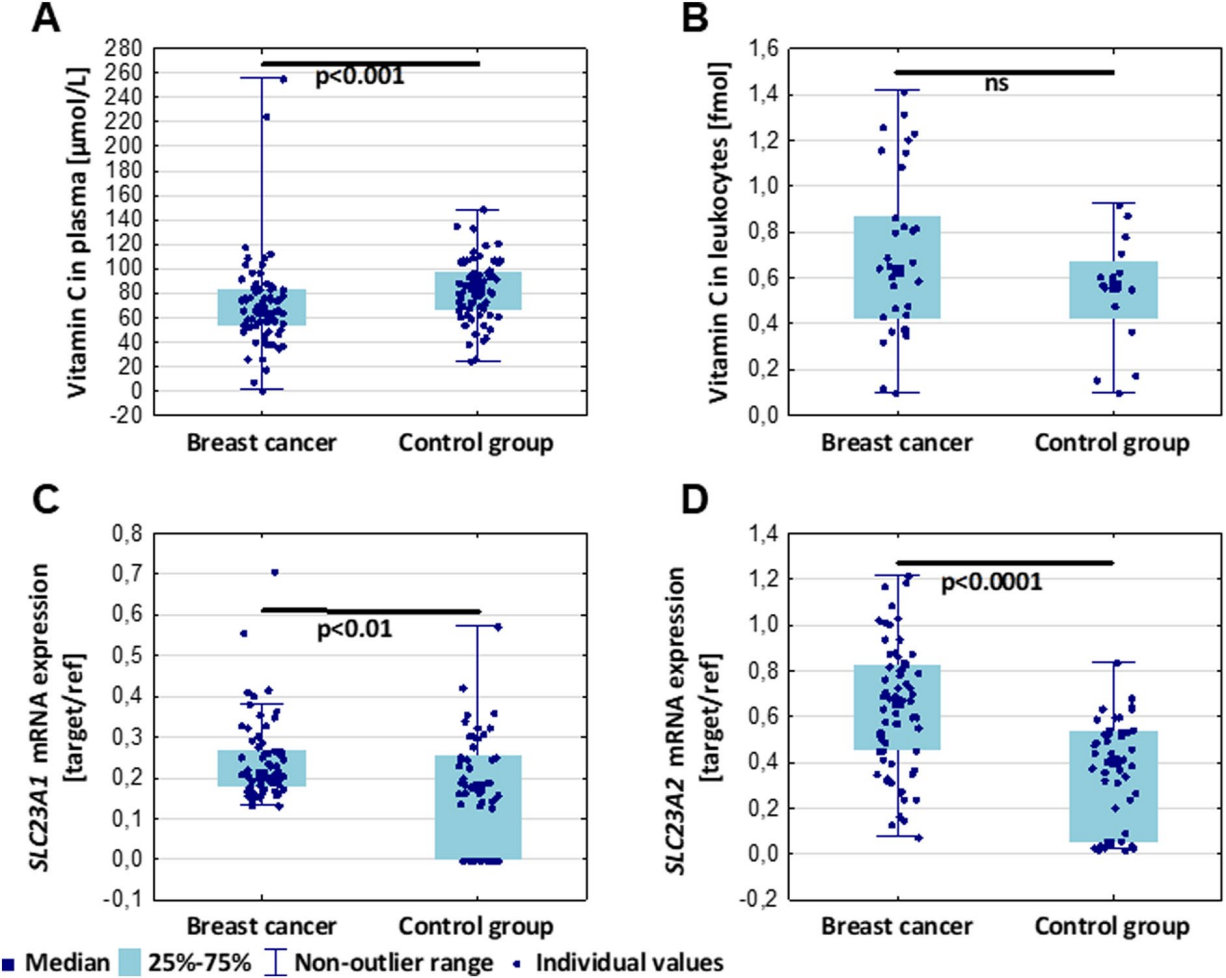
Comparison of vitamin C concentration in plasma (**A**) and in leukocytes (**B**), as well as SLC23A1 (**C**) and SLC23A2 (**D**) mRNA expression in leukocytes between breast cancer patients and control group. Plasma level of vitamin C was significantly elevated in the control group (**A**), whereas its level in leukocytes remained similar between breast cancer patients and healthy subjects (**B**). The expression level of both vitamin C transporters (SLC23A1 **C** and SLC23A2 **D**) was increased in breast cancer patients in comparison to the control group. ns – non-statistical difference.

The most striking result to emerge from the data is fluctuations of vitamin C, indicating that its level in plasma differs significantly in breast cancer patients (Fig. 3). Interestingly, we did not find a significant difference in vitamin C level in leukocytes between breast cancer patients and healthy subjects. Moreover, mRNA expression of *SLC23A1* and *SLC23A2* was elevated in leukocytes from breast cancer patients (Fig. 3).

### Luminal B breast cancer shows a significant increase of TET3 mRNA expression and a decline in 5-caC level in blood samples (leukocytes)

**Figure 4:**
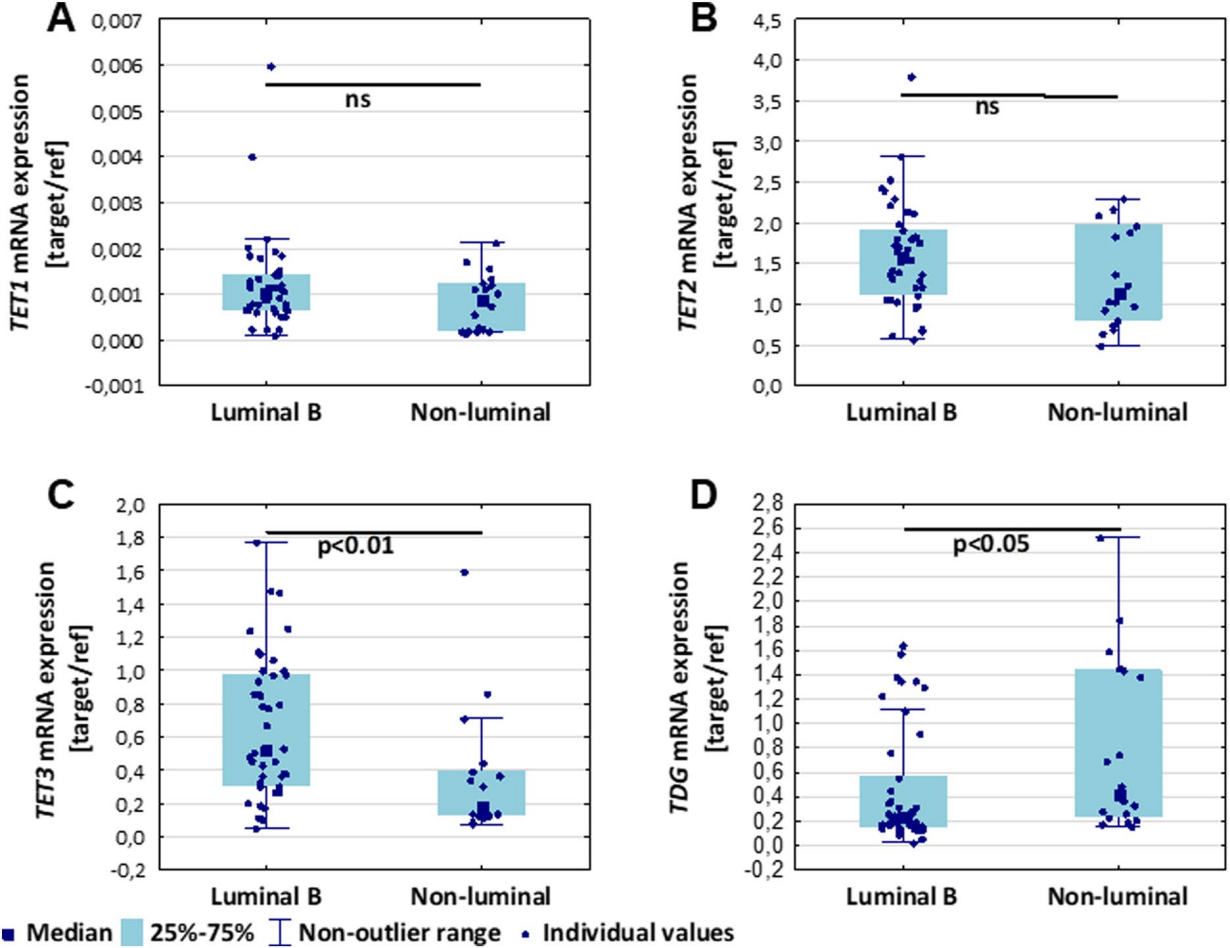
Comparison of TET1, TET2, TET3, and TDG mRNA expression between luminal B and non-luminal breast cancer patients. The expression levels of TET1 (**A**) and TET2 (**B**) were not statistically different between these two groups. Luminal B breast cancer group showed significantly higher TET3 mRNA expression (**C**), and decreased TDG mRNA expression (**D**) in comparison to non-luminal one. The expression level was presented as a relative value normalized by ACTB (β–actin, gene) and G6PD (glucose–6–phosphate dehydrogenase gene). ns – non-statistical difference.

Since breast cancer is a heterogeneous disease, we tried to compare changes between different biological types of breast malignancy. Amongst patients, who were recruited to the study only two groups (luminal B and non-luminal breast cancer groups) were large enough to perform statistical analyses. Our study indicated that luminal B and non-luminal breast cancers mRNA expression levels vary in terms of *TET3* and *TDG*. Luminal B breast cancers showed significantly higher *TET3* expression (*p* < 0.01), whereas *TDG* mRNA expression was increased amongst patients with non-luminal breast cancer subtype (*p* < 0.05) (Fig. 4C, D), despite scattered results the difference was statistically significant.

**Figure 5:**
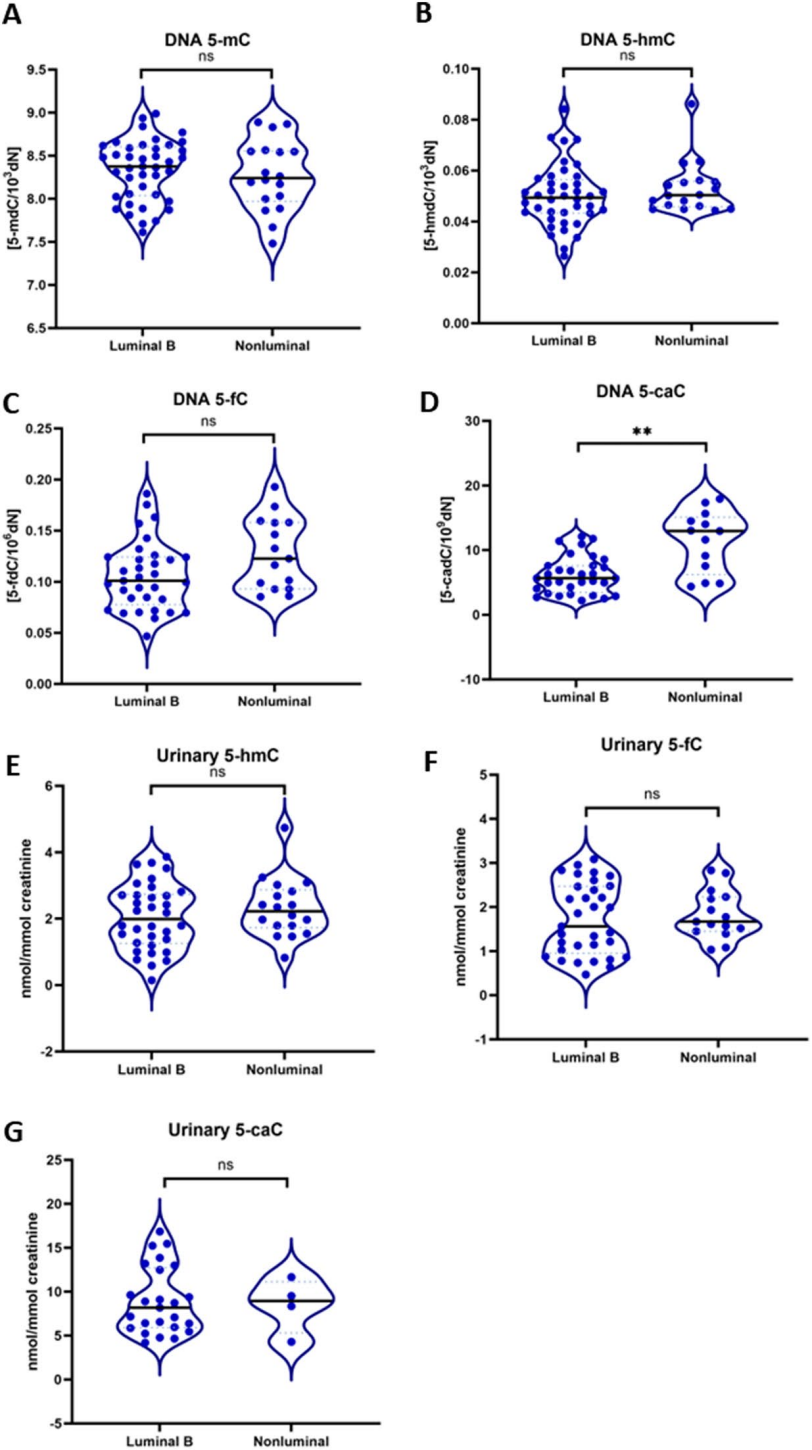
Comparison of 5-mC, 5-hmC, 5-fC and 5-caC in blood (DNA isolated from leukocytes) (**A**, **B**, **C**, **D**) and in urine (**E**, **F**, **G**) samples of luminal B and non-luminal breast cancer patients. 5-mC (**A**), 5-hmC (**B**) and 5-fC (**C**) were on similar levels in both luminal B and non-luminal groups. Luminal B breast cancer leukocytes showed significant decrease in 5-caC level (**D**). The 5-mC and its derivatives levels were presented as a level of modified bases to unmodified deoxynucleosides, which was expressed as a number of modified molecules per 10^–3^ (for 5-mC and 5-hmC), 10^–6^ (for 5-fC) or 10^–9^ (for 5-caC) of unmodified deoxynucleosides. No differences were noticed in urinary level of 5-hmC (**E**), 5-fC (**F**), or 5-caC (**G**) in both groups. 5-hmC and its derivatives in urine were presented as a relative value based on concentration of urinary creatinine. **p < 0.01; ns-non – statistical difference.

Levels of epigenetic modifications in DNA were comparable in terms of 5-mC, 5-hmC, and 5-fC (Fig. 5A–C). Only 5-caC level was significantly increased in non-luminal breast cancer in contrast to luminal B type (Fig. 5D). Epigenetic modifications’ levels in urine in non-luminal type corresponded with those in luminal B type (Fig. 5E–G).

We did not find any difference in vitamin C level between those two breast cancer types, both in plasma and leukocytes (Table 1).

**Table 1:** Comparison of vitamin C in plasma, vitamin C in leukocytes, SLC23A1 and SLC23A2 mRNA expression in leukocytes of luminal B and non – luminal breast cancer patients (ns—non statistical difference).

	Luminal B	Non-luminal	*p*
	Median (Q25-Q75)		
*Vitamin C in plasma*	66.3007 (54.2463–81.4594)	72.4207 (59.2726–83.2692)	ns
*Vitamin C in leukocytes*	0.6806 (0.4545–1.1542)	0.4860 (0.2393–0.6121)	ns
*SLC23A1 mRNA expression*	0.2079 (0.1669 -0.2664)	0.1974 (0.1816–0.2633)	ns
*SLC23A2 mRNA expression*	0.6674 (0.4549–0.8313)	0.5988 (0.4563–0.7092)	ns

## Discussion

Epigenetic regulation in the genome has been considered mainly in the context of mechanism which takes a leading role in cell development and differentiation, in processes such as genome imprinting or chromosome X inactivation^55^. Later, it was emerged that epigenetic processes may also play a crucial role in cancer initiation and progression. Several studies produced estimates of epigenetics’ aspect in the development of colon cancer, lung cancer or acute myeloid leukemia^56–58^. In reviewing the literature, it appears that changes in DNA methylation pattern, which may activate oncogenes or silence tumor suppressor genes, are important issues of epigenetic processes.

The milestone in understanding the dynamic changes in epigenome was the discovery of TET proteins more than a decade ago. Although, in recent years there has been an increasing interest in TETs mutations in different cancers, little did we know about their characteristic expression profile. Therefore, one of the aims of our study was the evaluation of TETs mRNA expression in leukocytes from breast cancer patients. A few previous studies have reported some variations of TETs expression in breast cancer patients. According to Yang et al. the expression of all TETs is decreased in primary breast cancer tissues^59^. However, Sasidharan Nair et al.^60^ reported that *TET2* and *TET3* mRNA expression levels were increased in breast tumor tissue samples. Tissue Cancer Genome Atlas (TCGA) database study also revealed that TET3 expression is higher in triple negative and hormone-dependent breast cancer in comparison to control tissue^61^. Moreover, based on Gene expression omnibus (GEO), TCGA, and Genotype-Tissue Expression (GTEx) databases, elevated TET3 expression is associated with poor prognosis in ovarian cancer^62^. Another study, which concerns peripheral blood samples of breast cancer patients has revealed the increase in only TET2 and TET3 expression^63^. In line with the last finding, we have shown that leukocyte expression level of *TET3* is higher in breast cancer patients than in the control group. Moreover, *TET3* expression was lower in leukocytes from patients with more severe grade of breast cancer (p=0.045). Furthermore, leukocyte levels of 5-mC and 5-hmC are significantly lower in patients with breast cancer. Such alterations may be linked with possible different role of 5-hmC regulation by TET proteins^64^.

One of the most interesting observations in our study was significantly higher expression of *TET3* in luminal B breast cancer patients in comparison to non-luminal HER2 positive breast cancer patients as well as in healthy subjects. Previously, one in vitro study revealed that hormone – dependent MCF-7 cell line expressed a higher level of TET3 in comparison to control cells^65^. Our observation can be linked with potential role of estrogens in the modulation of *TET3* expression. As it was indicated in Guan et al. study^66^, there are unambiguous interactions between TET3 and estrogen receptor α (ERα). In vitro studies demonstrated that estrogen alone or in combination with progesterone can impact on increase of TET3 expression in human endometrial epithelial cells (HES)^67^, as well as in increase of TET3 protein in endometrial adenocarcinoma cell lines (AN3)^68^. Furthermore, in our study leukocyte levels of 5-mC and 5-hmC are significantly lower in patients who suffer from breast cancer. Such alterations may be linked with possible different role of 5-hmC regulation by TET proteins. Some evidence from experimental studies has implied that all TET proteins are involved in hydroxylation of 5-mC to 5-hmC, but the further stages of this process are driven only by TET2 and TET3^64,69,70^. Based on the results presented in the manuscript, breast cancer leukocytes were characterized by increased mRNA expression of TET3 and decreased level of 5-hmC. Therefore, there’s a significant, however, slight, negative correlation (R=−0.181606, *p*<0.05) between these two parameters (data not shown). Based on this preliminary data, we hypothesize that increased TET3 expression may result in increased hydroxylation of 5-mC through 5-hmC up to 5-caC, depleting the pool of 5-mC, and also 5-hmC, 5-fC, 5-caC, as a result of efficient DNA repair pathway (observed as the increased level of above-mentioned modifications in urine).

There is also a general agreement that 5-hmC is decreased in cancers, and according to systematic review by Chen et. al., lower levels of this derivative correlate with cancer progression^71^. Lower 5-hmC may be also explained by a significant decrease of vitamin C in the plasma of breast cancer patients. Vitamin C is a key compound which boosts the reaction catalyzed by TET proteins. Ascorbic acid can bind to the catalytic domain of TET and restore the oxidation state of the iron ion, which is located in a catalytic center of enzyme^33^. However, the interaction between TET proteins and vitamin C is not limited to ascorbic acid reducing ability, since other oxidant agents do not present similar activity with TETs^72^. Thus, it seems possible that lower vitamin C level can impair TET functions, and consequently decrease 5-hmC level. It has been proven in our study: we have observed lower level of vitamin C in plasma of breast cancer patients in comparison to healthy subjects. Interestingly, we found higher mRNA expression of vitamin C transporters (*SLC23A1* and *SLC23A2*) in breast cancer patients in comparison to control samples. This observation is consistent with previous studies where SVCT2 was not immunohistochemically detectable in normal breast cells in comparison to cancer ones^73^. High expression of vitamin C transporters may increase the uptake of vitamin C in plasma followed by its decreased levels. This hypothesis may be supported by our observations that vitamin C level in leukocytes of breast cancer patients was similar to the level of this compound in leukocytes of healthy subjects. As a matter of fact, studies on mice incapable of synthesizing vitamin C in vivo revealed the increase in *SVCT* mRNA expression level in liver^74,75^, cerebellum^75^ or intestine^76^. Moreover, in vitro study demonstrated that breast cancer cell lines transport vitamin C via SVCT2 transporter^77^. Thus, high level of vitamin C transporters may compensate intracellular vitamin C level in breast cancer patients observed in our study.

5-mC and 5-hmC are stable cytosine modifications, their contents in the human genome many fold higher exceed the amount of other derivatives: 5-fC and 5-caC. The amount of 5-fC has estimated for 20 × 10^−6^ % cytosines, and 5-caC for 3 × 10^−9^ % cytosines in embryonic mice cells^78^. The main reason for such a scarce abundance of those derivatives in DNA may proceed from their instability and fast removal by effective DNA repair mechanisms which, in turn, leads to demethylation. The disturbed balance between methylation and demethylation processes, which is one of the hallmarks of carcinogenesis, may be involved in accumulation of 5-fC and 5-caC. However, despite that, analysis of further 5-hmC derivatives has become an urgent scientific issue lastly, there are scarce studies concerning the quantitative amount of 5-fC and 5-caC in cells, especially cancer ones. Chowdhury et al. found no differences in 5-caC between leukocytes from lung and pancreatic cancer and control group^79^. In turn, Eleftheriou et. al. observed a higher level of 5-caC in breast tumor tissues, however, it was a very small group and the 5-caC level was entirely not detected in histopathologically unchanged counterparts^80^. Similarly, the increased level of 5-caC, but also 5-fC, was noticed in prostate cancer tissue in comparison to controls^19^. In the aforementioned studies, the level of 5-fC and 5-caC was measured by immunohistochemistry methods. In the presented paper, the level of 5-hmC derivatives was assessed using highly sensitive and highly specific isotope dilution two-dimensional ultra-performance liquid chromatography with tandem mass spectrometry detection. We found a decrease of 5-fC and 5-caC level in leukocytes of breast cancer patients in comparison to healthy subjects. The observed decrease in the level of 5-caC may be the effect of altered TETs activity. Furthermore, a recent study has emerged for possible hydroxylation of 5-mC to 5-fC and 5-caC with omitting 5-hmC, which can be driven by TET2 protein. Such a mechanism implies that all potential 5-mC transformations are thermodynamically possible in terms of biochemical processes^81^ and it may explain the low level of 5-fC in leukocytes of breast cancer patients. There is, however, another plausible explanation: several studies reported that the 5-hmC stage may be potentially regulated in the active DNA demethylation process. It was suggested that TET proteins have various affinity to particular 5-mC derivatives, by leading to inhibition of further oxidation of 5-hmC and consequently decrease of 5-fC and 5-caC, or by its iterative process causing opposite effect^82,83^. Furthermore, some evidence has emerged that each epigenetic cytosine modification is potentially prone to react with particular proteins, which may determine their fate in active DNA demethylation or other epigenetic processes^84^.

The study revealed that 5-caC level was lower in DNA isolated from leukocytes of breast cancer patients. Interestingly, the urine level of 5-caC in this group was significantly increased, while 5-fC was comparable to control. These results can be explained in part by the association between the formation and elimination of 5-fC and 5-caC. It seems possible that there is a peculiar proportionality in 5-mC derivatives levels in DNA and urine: the rate of particular derivative elimination is dependent on its formation: the greater the excretion of 5-caC derivative in urine is, the lower level of 5-caC in DNA is. In turn, no significant differences in 5-fC in urine may be connected with its transformation to 5-caC which is excreted in higher level in urine.

TDG plays a leading role in identification and removal of 5-fC and 5-caC from DNA, which triggers the other components of BER^5,85^. The research conducted on mice model reveals that loss of *Tdg* gene is associated with embryos lethality, which may prove the crucial role of TDG in cellular development and maintenance of correct DNA methylation pattern, and in turn, epigenetic stability^86,87^. Moreover, it was also suggested that TDG has an ability to act like a tumor suppressor gene. The high TDG expression is associated with transcriptional activity of coactivators of p53 family proteins^88^. The p53 protein itself may potentially modulate the TDG expression through binding with TDG promoter and regulating its nuclear translocation^89^. In line with this hypothesis, high TGD level was observed in patients with colorectal cancer^90^. According to the literature, TDG level can be also involved with patients’ prognosis: Yang et. al. revealed that lower TDG expression is correlated with higher stage and size of breast tumors^91^.

Contrary to the expectations, in the presented study we did not find a significant difference in TDG expression level between breast cancer patients and healthy subjects. One question that needs to be addressed, however, is whether TDG expression has an influence on actual 5-fC and 5-caC production and excretion with urine. There is a common belief that the presence of the modifications in the urine primarily represents the repair product of DNA damage in vivo and reflects the activity of repair pathways. Therefore, the most plausible source of the modifications excreted in the analyzed urine is the DNA repair process. The excretion of epigenetic DNA modification into urine is equal to the rate of active demethylation of DNA. The increase in the urinary excretion of 5hmC observed in our study may represent alterations in the rate of DNA repair. Based on our results that TDG expression in breast cancer patients corresponds with its level in controls we hypothesize TDG might not affect the level of 5-fC and 5-caC at gene’s level. However, further studies are needed to thoroughly elucidate that issue.

Although, the study found no significant differences in TGD level between the whole group of breast cancer patients and healthy subjects, patients with non – luminal HER2 positive breast cancer had significantly higher TDG level than patients with luminal B type. It should be remembered that TDG can possibly mediate in transcription of different genes and regulating factors. TDG protein can directly interact with estrogen receptor ERα and moderate its location in the promoter of TFF1 gene, which encodes presenilin 2 (pS2)^92^ protein found generally in breast cancer cells^93,94^. Thus, a high expression of TDG correlates with increase in a TFF1 expression. Additionally, it was proven that in the course of TFF1 promoter activation there is cyclical methylation and demethylation. TDG and DNMT3a and DNMT3b proteins play the main roles in this process, which participate in DNA demethylation by 5-mC deamination^95,96^. A plausible alternative mechanism of DNA demethylation by 5-mC deamination depended on estrogen receptor was observed in breast cancer cell lines’ study, which may imply a key role of TDG in estrogen dependent signaling in breast cancer cell lines^97^.

## Conclusion

The presented paper demonstrated for the first time the global analysis of active DNA demethylation process, both expression of genes, and level of cytosine derivatives involved in this cycle in breast cancer patients, as well as compounds that affect this process such as vitamin C. Our research proved the differences between breast cancer patients and healthy subjects in leukocytes. The increase of *TET3* expression was concomitant with a reduced level of 5-hmC. Hence, it could be suggested that *TET3* expression and 5-hmC level may have the potential to serve as prognostic markers of breast cancer. Moreover, our study shed new light on potential estrogen/progesterone modulation of TET3 as it was significantly increased in luminal B breast cancer patients. Additionally, the low 5-hmC level in leukocytes of breast cancer patients in comparison to leukocytes of control group, implies the possible 5-hmC role as a diagnostic tool in cancer detection. Furthermore, other significant findings to emerge from this study are differences of plasma vitamin C levels. The observed differences in vitamin C levels may indicate some disturbances in cellular transport of vitamin C or metabolic changes which can affect its elimination.

## Methods

### Study group

The study group included blood and urine samples from 74 female breast cancer patients (median age 55 ± 10 years) (Table 2). None of the study subjects were related with one another, and all of them were Caucasians. All participants of the study were recruited in a hospital setting (Professor Franciszek Lukaszczyk Oncology Centre, Bydgoszcz, Poland) during the first diagnostic appointment. The study was conducted in accordance with the Declaration of Helsinki, its protocol was approved by the Local Bioethics Committee at Collegium Medicum, Nicolaus Copernicus University in Bydgoszcz, Poland (KB 806/2015), and written informed consent was obtained from all the subjects. Blood and urine samples were taken at the moment of diagnosis based on histopathological examination from a core needle biopsy (CNB), hence therapy did not impact the measured parameters. The patients were classified by: age (n = 46 (62%) were after 50 year of age), BMI (n = 27 (36%) were overweight, and n = 21 (28%) had normal body weight), histopathological diagnosis (n = 64 (86%) developed invasive carcinoma of no special type (NST)), clinical stage (n = 43 (58%) were in the II stadium of clinical stage), and biological subtype (n = 42 (57%) developed luminal B subtype, and n = 18 (24%) were diagnosed as non-luminal HER2 positive subtype).

**Table 2:** The characterization of breast cancer patients involved into the study.

Clinical characteristic	Breast cancer (n = 74)
Age	55 ± 10 years
> 50 years	46 (62%)
< 50 years	26 (35%)
* No data*	*2 (3%)*
BMI	Average 27,7
Normal (BMI 18,5–24,99)	21 (28%)
Overweight (25–29,99)	27 (36%)
Obese class I (30–34,99)	13 (18%)
Obese class II (35–39,99)	5 (7%)
Obese class III (> 40)	1 (1%)
* No data*	*7 (10%)*
Histopathological result	
NST invasive cancer	64 (86%)
Sarkoid carcinoma	1 (1%)
Invasive lobular carcinoma	3 (4%)
Preinvasive ductal carcinoma	1 (1%)
Mucous carcinoma	1 (1%)
NST invasive cancer with preinvasive ductal carcinoma	1 (1%)
NST invasive cancer with mucous cancer	1 (1%)
* No data*	*2 (3%)*
Size of primary tumor (TNM)	
T1	5 (7%)
T2	42 (57%)
T3	6 (8%)
T4	17 (23%)
* No data*	*4 (5%)*
Degree of spread to regional lymph nodes (TNM)	
N0	29 (39%)
N1	30 (41%)
N2	10 (14%)
N3	1 (1%)
* No data*	*4 (5%)*
Clinical stage	
I	2 (3%)
II	43 (58%)
III	25 (34%)
* No data*	*4 (5%)*
Molecular/ biological type	
Luminal A (HER2(-), ER/PR( +))	6 (8%)
Luminal B (HER( +), ER/PR( +))	42 (57%)
Non-luminal (HER2( +), ER/PR(-))	18 (24%)
Triple negative (HER2(-), ER/PR(-))	6 (8%)
* No data*	*2 (3%)*
Degree of remission	
Partial remission	30 (41%)
Entire remission	31 (42%)
Patients during treatment	13 (18%)

The control group included 71 females without any oncological treatment history, who were not diagnosed with breast cancer (median age 53 ± 10 years). The median BMI was 27.7 in breast cancer group, while in control group 26.3. Statistical analysis did not show any significant intergroup differences in terms of age and BMI.

### Isolation of leukocytes’ DNA and determination of epigenetic modifications in DNA isolates

Leukocytes from heparinized blood samples were isolated using Histopaque 1119 solution (Merck) as per the manufacturer’s recommendations, following the storage in – 80 °C until analysis. DNA from leukocytes was isolated using the method described earlier^98,99^. See supplementary material. Briefly, leukocytes were resuspended in ice-cold buffer B (10 mM Tris–HCl (Merck KGaA, Germany), 5 mM Na2EDTA (Merck KGaA, Germany) and 0.15 mM deferoxamine mesylate (Merck KGaA, Germany), pH 8.0) in a 1:1 ratio. Next, SDS (Merck KGaA, Germany) was added (final concentration of 0.5%), and samples were incubated at 37 °C for 30 min, followed by proteinase K (Merck KGaA, Germany) addition (final solution concentration 4 mg/mL) and incubation at 37 °C for another 1.5 h. Next using phenol: chloroform: isoamyl alcohol (25:24:1) in a 1:1 ratio, samples were extracted, and the aqueous phase was treated with a chloroform: isoamyl alcohol mixture (24:1). Next, the supernatant was treated with cold 96% (v/v) ethanol to precipitate high molecular weight nucleic acids. The obtained precipitate was dissolved in Milli-Q grade deionized water. The samples were mixed with 200 mM ammonium acetate containing 0.2 mM ZnCl2, pH 4.6 (1:1). Nuclease P1 (100 U, New England Biolabs) and tetrahydrouridine (Merck KGaA, Germany), 10 μg/sample was added to the mixture and incubated at 37 °C for 3 h. Subsequently, 10% (v/v) NH4OH and 6 U of shrimp alkaline phosphatase (rSAP, New England Biolabs) was added to each sample and incubated for 1.5 h at 37 °C. Finally, all the hydrolysates were ultrafiltered prior to injection to eliminate macromolecular compounds, using AcroPrep Advance 96-Well Filter Plates 10 K MWCO (Pall Corporation, USA) and centrifugation at 2000 × g for 60 min at 4 °C.

The quantification of 5-methyl-2’-deoxycytidine (5-mdC), 5-(hydroxymethyl)-2’-deoxycytidine (5-hmdC), 5-formyl-2’-deoxycytidine (5-fdC), and 5-carboxy-2’-deoxycytidine (5-cadC) by 2D-UPLC-MS/MS was performed by the method reported in the previous paper. Briefly the molar concentration of modified deoxynucleoside was divided by the sum of molar concentrations of unmodified deoxynucleosides (dN), which served as “secondary internal standard”, and has been expressed as the number of modified molecules per thousand (5-mC and 5-hmC), million (5-fC), or billion of unmodified deoxynucleosides (5-caC), depending on their abundance^98,100^.

### RNA isolation and gene expression analysis

RNA isolation was carried out with PAXgene™ Blood RNA kit (Qiagen) according to Manual Purification of Total RNA from Human Whole Blood Collected into PAXgene Blood RNA Tubes protocol. The quality and integrity of the total RNA were assessed by visualization of the 28S/18S/5.8S rRNA band pattern in a 1.2% agarose gel. Electrophoreses were carried out at 95 V for 20 min in TBE buffer (Tris – Boric Acid – EDTA). 0.5 μg of total isolated RNA from each sample in volume 20 μl were used for cDNA synthesis by reverse transcription using the High–Capacity cDNA Reverse Transcription Kit (Applied Biosystems) according to the procedures of producer on Mastercycler Nexus Gradient thermocycler (Eppendorf). To ensure the absence of genomic DNA contamination, negative controls were included in the reverse transcriptase reaction.

The qRT–PCR complied with the Minimum Information for Publication of Quantitative Real–time PCR Experiments (MIQE) guidelines. Gene transcripts – *TET1, TET2, TET3, TDG, SLC23A1, SLC23A2* were analyzed by relative quantitative real–time RT–PCR (qRT–PCR) using relevant primers and short hydrolysis probes substituted with Locked Nucleic Acids from Universal Probe Library – UPL, see Supplementary information.

### Determination of epigenetic modifications in urine

Two-dimensional ultra-performance liquid chromatography with tandem mass spectrometry (2D UPLC–MS/MS) method was used for epigenetic modifications analysis in urine (except 5-hmUra). Urine samples (2 mL) were spiked with a mixture of internal standards in 4:1 volumetric ratio. 2D-UPLC − MS/MS system consists of the gradient pump and autosampler for the first-dimension chromatography and the gradient pump and tandem quadrupole mass spectrometer with unispray ion source for the second-dimension chromatography^99,101^. See Supplementary information.

### Assessment of ascorbic acid concentration

#### Leukocytes preparation and determination of cell number

See Supplementary information.

#### Determination of intracellular vitamin C in leukocytes by UPLC-MS, described in^102^

See Supplementary information.

### Statistical analysis

All statistical analyses were performed with Statistica 13.1 PL software (Dell, Inc. (2016) Dell Statistica, version 13). Graphs were prepared using GraphPad Prism Software version 10.1.2 for Windows (GraphPad Software, Boston, Massachusetts USA) and Statistica 13.1 PL software (Dell, Inc. (2016) Dell Statistica, version 13).

We carried out regression and correlation analysis to investigate how data points are dependent. We also checked normality and lognormality tests (namely D’Agostino and Pearson, Anderson–Darling, Shapiro–Wilk, Kolmogorov–Smirnov), which indicated non-parametrical distribution of variables. Based on the results, we decided to use the corresponding test, Mann–Whitney or Student’s t- test (*p < 0,05, **p < 0,01, and ***p < 0,001 indicate statistical significance, ns non-statistical significance *p* > 0,05), since we were comparing leukocytes from luminal B and non-luminal breast cancer groups. The results are presented as medians, interquartile, non-outlier range and individual. . Correlations between variables were evaluated based on Spearman correlation coefficients for raw data.

### Ethics approval and consent to participate

The study was conducted in accordance with the Declaration of Helsinki, its protocol was approved by the Local Bioethics Committee at Collegium Medicum, Nicolaus Copernicus University in Bydgoszcz, Poland (KB 806/2015), and written informed consent was sought from all the subjects.

### Supplementary Information

Supplementary Information.

## Data Availability

The datasets used and/or analyzed during the current study are available from the corresponding authors on reasonable request.
