# Supplementary material for: The level of active DNA demethylation compounds in leukocytes and urine samples as potential epigenetic biomarkers in breast cancer patients
Source: Sci Rep. 2024 Mar 18;14:6481. doi: 10.1038/s41598-024-56326-5 (PMC10948817; doi:10.1038/s41598-024-56326-5)
Supplement: Supplementary file 1 — Supplementary Information. [file 41598_2024_56326_MOESM1_ESM.pdf]

Supplementary information

Methods

RNA isolation and gene expression analysis

The probes are labeled at the 5'end with fluorescein (FAM) and at the 3'end with a dark quencher dye. The expressions of target genes were normalized relatively to two reference genes ACTB (β–actin, Roche gene ID: 60), and G6PD (glucose–6–phosphate dehydrogenase, Roche gene ID: 2539). The real–time PCR mixes in the volume 10μl were prepared from cDNA preparations according to the standard procedures of LightCycler480 Probes Master provided with the reagents set. Quantitative real time PCR was carried out by LightCycler 480 II instrument with the following cycling parameters: 10s at 95°C followed by 45 repeats of 10s at 95°C, 30s at 58°C, and finally 1s at 72°C with acquisition mode (wavelength excitation: 465nm for target genes, and 533nm for reference genes; wavelength detection: 510nm for target genes and 580nm for reference genes). The standardizations of reaction for each gene were performed to estimate the efficiency of amplification via standard curves.

Supplementary table 1. Primers and short hydrolysis probes used for *TET*s, *TDG*, *SLC23A1* and *SLC23A2* mRNA expression analysis.

Gene	Forward primer sequence	Reverse primer sequence	UPL
TET1	5’-TCTGTTGTTGTGCCTCTGGA-3’	5’-GCCTTTAAAACTTTGGGCTTC-3’	#57
TET2	5’-GCCTTTGCTCCTGTTGAGTT-3’	5’-ACAAGGCTGCCCTCTAGTTG-3’	#38
TET3	5’-CACTCCGGAGAAGATCAAGC-3’	5’-GGACAATCCACCCTTCAGAG-3’	#1

TDG	5'-GAATGGAAGCGGAGAACG-3'	5'-TTGCTGTTCATTCACAACTGC-3'	#41
SLC23A1	5'- GGGATTTTCCATGTTCTTCG-3'	5'- AATCTGATCCACTTCAAGAATGC-3'	#44
SLC23A2	5'- CAGCTGTTCAAAATGTTCCCTA-3'	5'- GTCACCGTGAAGATGAAGCA-3'	#71

Determination of epigenetic modifications in urine

Both dimensions are coupled with a column manager equipped with two programmable column heaters and two 6-port switching 2-position valves. At-column dilution technique was used between the first and the second dimension to improve the retention at a trap/transfer column. The sample molecules were then adsorbed to the packing material as very narrow bands that could be eluted as well-resolved, small-volume peaks. A diluting stream of water (0.5 mL/min) was pumped with Waters 515 isocratic pump and mixed with the first-dimension column effluent using a UPLC low-dead-volume tee. The following columns were used: CORTECS UPLC T3 Column (1.6 μm, 3 mm × 150 mm) with CORTECS T3 VanGuard Pre-column (1.6 μm, 2.1 mm × 5 mm) for the first dimension, Waters ACQUITY UPLC CSH C18 (1.7 μm, 2.1 mm × 100 mm) for the second dimension, and Waters XSelect CSH C18 Column (3.5 μm, 3 mm × 20 mm) as the trap/transfer column. The chromatographic system operated in a heart-cutting mode which means that selected portions of effluent from the first dimension were directed to the trap/transfer column via the 6-port valve switching which served as an "injector" for the second dimension chromatography system. Mass spectrometric detection was conducted with Waters Xevo TQ-S tandem quadrupole mass spectrometer equipped with

a unispray ionization source. The following common detector parameters were used: the source temperature 150°C, nitrogen desolvatation gas flow 1000 L/h, nitrogen cone gas flow 150 L/h, desolvatation temperature 500°C, and nebulizer gas pressure 7 bar. Collision-induced dissociation was obtained with argon (6.0 at 3 x 10-6 bar pressure) as collision gas. The instrument's response to all compounds was optimized by the infusion of 10 µM genuine compounds dissolved in water (10 µL/min), in mobile phase A stream, via the mass spectrometer fluidics system operating in the "mixed" mode, using MassLynx 4.1 Intelli-Start feature. The chromatographic system was operated with MassLynx 4.1 Software from Waters. Quantitative analyses were performed using Target Lynx application. All samples were analyzed in three to six technical replicates. Due to low sensitivity of the hereby used method the level of 5-hmUra was determined by high-performance liquid chromatography prepurification followed by gas chromatography with isotope dilution mass spectrometric detection (LC/GC–MS), as previously described [1].

Assessment of ascorbic acid concentration

Leukocytes preparation

Leukocytes suspended in 200 µL RPMI (Merck KGaA, Germany) were homogenised using an ultrasonic homogeniser (SONOPULS UW 2070, BANDELIN electronic GmbH & Co. KG), twice for 10 s on ice. After homogenisation, samples were placed on ice, then appropriate amounts of the homogenate were taken to determine the intracellular concentration of vitamin C and the thymine content.

Determination of cell number

The exact number of cell content in leukocytes was estimated based on thymine content using the method described by Modrzejewska et al. [2], with some modifications. It was assumed that the average diploid cell contains 6.7 fmol of thymine. Briefly, 20 µL of homogenate was incubated at 130 °C for 1 h with 200 µL 2 M HCl (Merck KGaA, Germany) containing of 515 µM caffeine as an internal standard (Merck KGaA, Germany) in a sealed 2 mL glass vial. The cooled sample was completely dried under nitrogen (XcelVap, Biotage AB), dissolved in 100 µL of the Milli-Q grade deionised water and ultrafiltered prior to the injection. A 2 µL aliquot of the sample was chromatographed at a flow rate of 0.45 mL/min and 40 °C on CORTECS® UPLC T3 1.6 µm (3 x 150 mm) column coupled to Waters Acquity UPLC system with a photodiode array detector, using two solvents: A - 10 mM ammonium formate (pH 3.14) and B - acetonitrile, according to the following elution program: 0 - 0.1 min, isocratic, 0.1 % B; 0.1 - 2 min, linear gradient 0.1 % - 15 % B; 2 - 3 min, linear gradient 15 % - 50 % B; 3 - 3.5 min, isocratic, 50 % B; 3.5 - 3.51 min, linear gradient, 50 % - 0.1 % B. The chromatographic peak of thymine was monitored with a photodiode array detector at 254 nm and analysed with the MassLynx software.

Determination of intracellular vitamin C in leukocytes by UPLC-MS, described in [3]

Forty-five microliters of the sample were mixed with 50 µL of 10 % (m/v) trichloroacetic acid (Merck KGaA, Germany) and 5 µL of 100 µM stable isotope-labelled internal standard solution ([13C6] L-ascorbic acid-, Toronto Research Chemicals) and incubated for 20 min on ice. Then, the samples were vortexed and centrifuged at 24400 × g for 20 min at 4 °C. The supernatants were filtrated using AcroPrep Advance 96-Well Filter Plates 10 K MWCO (Pall Corporation, USA). One microliter of the aliquots was

chromatographically separated on a CORTECS® UPLC T3 1.6 µm (3 mm × 150 mm) column with a Waters Xevo TQ-XS tandem mass spectrometer. The column (20 °C) was eluted at a flow rate of 0.3 mL/min with 5 µM ammonium formate in 0.05 % acetic acid (solvent A) and methanol (solvent B). The electrospray ionisation was set to negative ion mode. The desolvation gas (nitrogen) flow rate was 1200 L/h, the nitrogen cone gas flow was 200 L/h, the desolvation temperature was 500 °C, and the nebuliser gas pressure was 7 bar. Collision-induced dissociation was obtained with argon (3×10-6 bar pressure) as the collision gas. Transition patterns that were selected as quantitative (175>115 and 181>119 for L-ascorbic acid and [13C6]-L-ascorbic acid, respectively) were acquired using MassLynx 4.2 software from Waters. Quantitative analyses were performed using the Target Lynx application. All the samples were analysed in three to five technical replicates**.** The quantification of 5-methyl-2'-deoxycytidine (5-mdC), 5-(hydroxymethyl)-2'-deoxycytidine (5-hmdC), 5-formyl-2'-deoxycytidine (5-fdC), and 5-carboxy-2'-deoxycytidine (5-cadC) by 2D-UPLC-MS/MS was performed by the method reported in the previous paper. Briefly the molar concentration of modified deoxynucleoside was divided by the sum of molar concentrations of unmodified deoxynucleosides (dN), which served as"secondary internal standard", and has been expressed as the number of modified molecules per thousand (5-mC and 5-hmC), million (5-fC), or billion of unmodified deoxynucleosides (5-caC), depending on their abundance [4,5].

1. Rozalski, R. *et al.* Urinary 5-hydroxymethyluracil and 8-oxo-7,8-dihydroguanine as potential biomarkers in patients with colorectal cancer. *Biomarkers* **20**, 287–291 (2015).

2. Modrzejewska, M., Gawronski, M. & Gackowski, D. Normalization of metabolic data to total thymine content and its application to determination of 2-hydroxyglutarate. *Anal Biochem* **618**, 114129 (2021).

3. Starczak, M. *et al.* Dynamic changes in genomic 5-hydroxymethyluracil and N6-methyladenine levels in the Drosophila melanogaster life cycle and in response to different temperature conditions. *Sci Rep* **12**, 17552 (2022).

4. Starczak, M., Gawronski, M., Olinski, R. & Gackowski, D. Quantification of DNA Modifications Using Two-Dimensional Ultraperformance Liquid Chromatography Tandem Mass Spectrometry (2D-UPLC-MS/MS). *Methods Mol Biol* **2198**, 91–108 (2021).

5. Gackowski, D. *et al.* Accurate, Direct, and High-Throughput Analyses of a Broad Spectrum of Endogenously Generated DNA Base Modifications with Isotope-Dilution Two-Dimensional Ultraperformance Liquid Chromatography with Tandem Mass Spectrometry: Possible Clinical Implication. *Anal Chem* **88**, 12128–12136 (2016).
